# The expression of the BPIFB4 and CXCR4 associates with sustained health in long-living individuals from Cilento-Italy

**DOI:** 10.18632/aging.101159

**Published:** 2017-01-22

**Authors:** Gaia Spinetti, Elena Sangalli, Claudia Specchia, Francesco Villa, Chiara Spinelli, Rita Pipolo, Albino Carrizzo, Simona Greco, Christine Voellenkle, Carmine Vecchione, Paolo Madeddu, Fabio Martelli, Annibale Alessandro Puca

**Affiliations:** ^1^ Cardiovascular Research Unit, IRCCS MultiMedica, 20138 Milan, Italy; ^2^ University of Brescia, 25121 Brescia BS, Italy; ^3^ Department of Medicine and Surgery, University of Salerno, 84084 Salerno, Italy; ^4^ Laboratory of Vascular Pathophysiology, IRCCS Neuromed, 86077 Pozzilli (IS), Italy; ^5^ IRCCS Policlinico San Donato, San Donato, Italy; ^6^ University of Bristol, Bristol BS28HW, UK

**Keywords:** long-living individuals, mononuclear cell migration, BPIFB4, HIF-1ɑ, targets, CXCR4

## Abstract

The study of the health status in long-living individuals (LLIs) may help identifying health-span and life-span determinants. BPI-Fold-Containing-Family-B-Member-4 (BPIFB4) protein is higher in healthy vs. non-healthy (frail) LLIs serum and its longevity-associated variant forced expression improves cardiovascular outcomes in ischemia mice models. Thus, we tested the association of *BPIFB4* and ischemia-responding HIF-1ɑ pathway components (i.e. *CXCR4*, *AK3, ALDO-C*, *ADM*, *VEGF-A*, *GLUT-1* and miR-210) with human life-span and health-span by analyzing mRNA expression in circulating mononuclear cells (MNCs) of LLIs (N=14 healthy; N=31 frail) and young controls (N=63).

*ALDO-C*, *ADM*, *VEGF-A* and *GLUT-1* significantly decreased and miR-210 increased in LLIs vs. controls. Only *VEGF-A* and *GLUT-1* showed further significant reduction in healthy-LLIs vs. frail-LLIs comparison. While *BPIFB4* and *CXCR4* were similar between LLIs and controls, *BPIFB4* was significantly higher and *CXCR4* lower in healthy- versus frail-LLIs. On a new set of LLIs (N=7 healthy and N=5 non-healthy) we assessed a potentially correlated function with low CXCR4 expression. Healthy donors' MNCs showed efficient migration ability toward CXCR4 ligand SDF-1ɑ/CXCL12 and high percentage of migrated CXCR4^pos^ cells which inversely correlated with CXCR4 RNA expression. In conclusion, BPIFB4 and CXCR4 expression classify LLIs health status that correlates with maintained MNCs migration.

## INTRODUCTION

The dramatic increase of life expectancy during the 20th century represents one of the greatest achievements of the human society, but also creates new challenges to the sustainability of current national health systems [1, 2]. The incidence and prevalence of age-related diseases, including cardiovascular diseases (CVD), increase exponentially with advancing age, both clinically and sub-clinically, and have an important impact on morbidity and mortality, imposing an extra-ordinary economic toll on individuals and public health budgets. Hence, it is urgent to introduce innovative approaches to extend and/or restore the period of youthful health of older people [3].

The rising life expectancy within the older population itself is increasing the number and proportion of very old people. The global number of centenarians is projected to increase 10-fold between 2010 and 2050. Long living individuals (LLIs), that are 90 years of age or older, represent a class of subjects that can provide the scientific community with an invaluable information about key determinants of human life span. Moreover, some LLIs are healthy, e.g. they escape age-associated diseases, while others are non-healthy, presenting or having a history of typical age-associated diseases. Thus, while comparison of LLIs versus young controls identifies genes associated with global longevity, the comparison of healthy-LLIs versus non-healthy-LLIs may provide fundamental insights into the mechanistic classifiers of the human health-span, and not aging itself. Nevertheless, the potential of the latter approach has not been thoroughly investigated.

We recently identified four missense mutations (that allow mutant recombinant proteins experiments) among the top findings (p<0.0001) of our recent genome-wide association study on LLIs and controls of Cilento-Italy, and replicated them in two independent populations from US and Germany. This investigation led to the identification of a significant enrichment in LLIs of homozygous genotypes of the minor allele of rs2070325 polymorphism (Ile229Val in *BPIFB4*). Strong linkage disequilibrium in BPIFB4 locus generated two alternative haplotypes (the WT Ile229/Asn281/Leu488/Ile494 and the longevity-associated-variant-LAV al229/Thr281/Phe488/Thr494; identifier: P59827.2). The LAV-BPIFB4 is phenotypically characterized by the activation of the endothelial nitric oxide synthase (eNOS) signalling pathway, leading to improvements in endothelial function, vascular reactivity to hypertensive stimuli, and regenerative capacity following ischemic insults [4]. BPIFB4 is a secreted serum protein and is also contained in circulating mononuclear cells. Its abundance may represent a classifier of healthy aging. In fact, we showed that high serum BPIFB4 protein levels identify healthy LLIs, distinguishing them from those affected by typical geriatric diseases [5].

Another powerful approach to unraveling the secret of longevity employs model organisms. Groundbreaking studies in the *Caenorhabditis elegans*, one of the principal models used to study aging, have shown that the hypoxia-inducible factor 1 (HIF-1) plays pivotal roles in the modulation of the regulatory networks that link oxygen homeostasis and aging [6, 7]. Under ischemic conditions, tissues are starved of nutrients and exposed to low oxygen levels, which trigger the HIF-1 transcription, thus enabling the activation of adaptive responses to stress. HIF-1 is a heterodimer consisting of one alpha unit (HIF-1α, HIF-2α or HIF-3α) and one beta subunit (HIF-1β) [8]. HIF-1 can activate the transcription of more than 200 genes, constituting a significant portion of the hypoxia-induced transcription. Of note, HIF-1 targets are not limited to protein coding transcripts, but can also regulate noncoding RNAs, including microRNAs (miRNAs) and long noncoding RNAs [9]. In particular, we and others identified miR-210 as a master “hypoxamiR” modulating cell metabolism and survival as well as angiogenesis [10]. By targeting, both directly and indirectly, several components of the oxidative phosphorylation mitochondrial machinery, miR-210 expression impacts on metabolism, contributing to the switch towards glycolysis observed in hypoxia [11]. Moreover, miR-210 expression in normoxic endothelial cells stimulates the formation of capillary-like structures, and VEGF-driven cell migration, at least in part, by targeting ephrin-A3 (EFNA3) adhesion molecule [12]. Additionally, miR-210 expressing cells display increased angiogenic factor signaling [13-15]. According with a pro-angiogenic role of miR-210, tissue perfusion and capillary density in mouse ischemic hindlimbs are both increased by the injection of CD34+ umbilical cord blood cells expressing miR-210 [16] and miR-210 overexpression induces angiogenesis in the normal adult mouse brain [17].

Circulating mononuclear cells (MNCs) are involved in vascular repair and regeneration, which led to the proposal of assessing the MNC trascriptome and function for an improved classification of cardiovascular reactivity to injury [18-21]. Thus, we hypothesize that the assessment of gene transcripts in MNCs may inform on the organism potential to maintain the cardiovascular homeostasis during aging. In the attempt to find genes/miRNAs associated with either life-span or health-span, we here conducted a gene expression analysis of BPIFB4 together with a set of RNAs under the control of HIF-1ɑ in peripheral blood MNCs from healthy-, non-healthy-LLIs and young controls. Results indicate that both BPIFB4 and a certain HIF-1ɑ-associated genes are powerful classifiers of the health status in LLIs.

## RESULTS

### Components of the BPIFB4 and HIF-1ɑ pathway classify healthy long-living individuals

RNA expression levels of BPIFB4 and components of the ischemia-responding HIF-1ɑ pathway (namely *CXCR4*, *AK3, ALDO-C*, *ADM*, *VEGF-A*, *GLUT-1* and *miR-210*) were measured in peripheral blood circulating MNCs. Few HIF-1ɑ targets were selected as an example of the multiple cell function controlled by this transcription factor, i.e. angiogenesis and survival (ADM, VEGF-A and miR-210), glucose metabolism (ALDO-C, GLUT-1), migration (CXCR4), and cellular homeostasis (AK3).

Table 1 shows the characteristics of the 45 LLIs and 63 controls analyzed in the differential expression analyses. Mean age was 97.4 years for LLIs, 44.1 years in the control group. Gender distribution was similar in the two groups (p=0.8). Among the LLIs, 31 (68.9%) were frail. The diagnoses included 5 cases of diabetes (16.1%), 10 CVD (32.3%), 3 diabetes and CVD (9.7%), 8 Alzheimer or dementia (25.8%), 1 Alzheimer and CVD (3.2%), 2 respiratory diseases (6.5%), 1 rheumatoid arthritis (3.2%) and 1 rheumatoid arthritis and CVD (3.2%).

**Figure 1:**
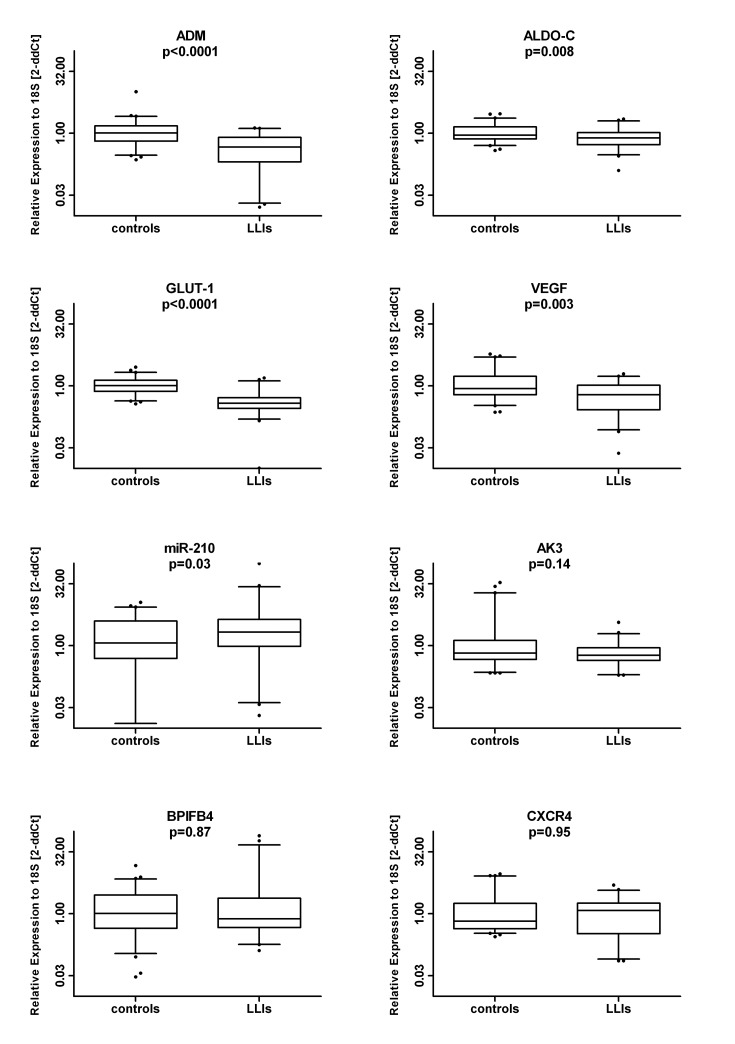
Circulating MNCs of long living individuals (LLIs) show similar BPIFB4 gene levels but modulation in HIF-1ɑ hallmark factors compared to young controls Box plots of the mRNA levels (log2 scale) of BPIFB4 and HIF-1ɑ-associated factors (CXCR4, ALDO-C, ADM, miR-210, VEGF-A, GLUT-1, AK3) in LLIs (N=45) versus controls (N=63).

In the comparison between the total LLI population and controls, we found a reduction of *ADM* (median (range): 0.46 (0.02-1.34) vs. 1.02 (0.23-10.18) p<0.0001), *ALDO-C* (median (range): 0.76 (0.12-2.21) vs. 0.90 (0.38-2.95) p=0.008), *GLUT-1* (median (range): 0.38 (0.01-1.56) vs. 1.01 (0.37-2.85) p<0.0001) and *VEGF-A* (median (range): 0.61 (0.02-1.95) vs. 0.86 (0.23-5.89) p=0.003) whereas miR-210 was increased (median (range): 2.13 (0.02-98.64) vs. 1.16 (0.01-11.32) p=0.03) (all data are expressed as 2ddCt relative expression values). In contrast, BPIFB4, CXCR4 and AK3 mRNA levels did not differ between the two groups (Figure 1).

**Figure 2:**
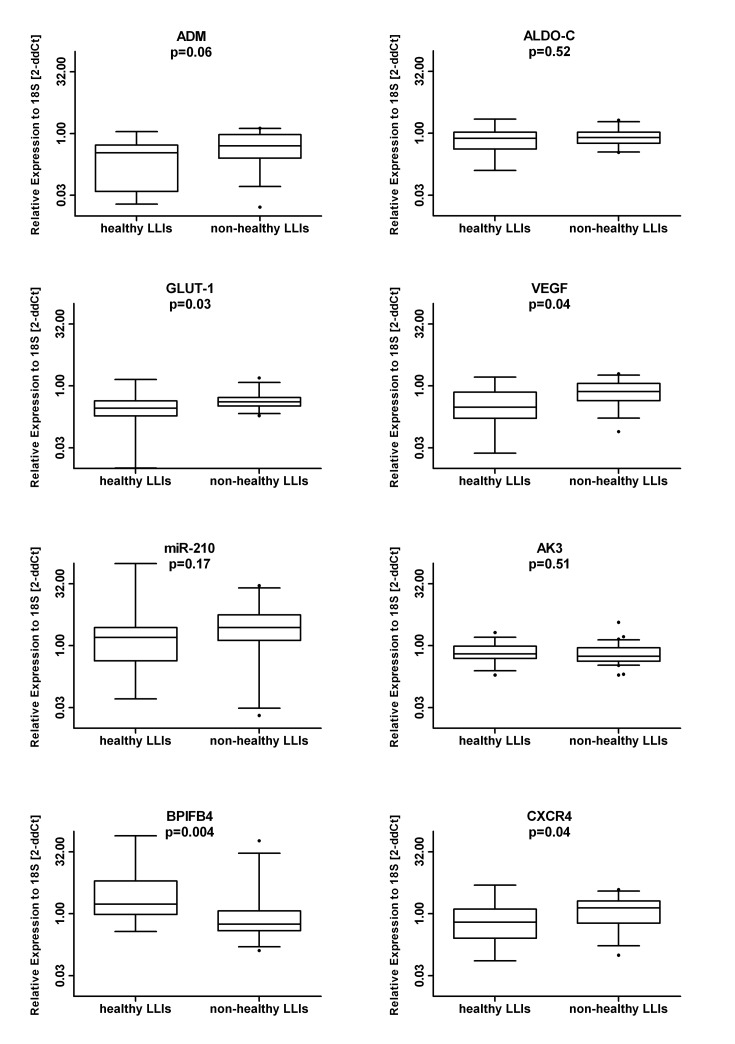
Effect of disease on the mRNA signature The LLI group was subdivided based on the presence/absence of age-associated diseases. Next, the RNA levels in MNC of the indicated genes was measured in healthy (N=14) vs. non-healthy (N=31) donors. Data are shown as box plots in a log2 scale.

When analyzing the LLIs cohort according to the health condition, healthy-LLIs showed significantly lower levels of *VEGF-A* (median (range): 0.30 (0.02-1.62) vs. 0.72 (0.77-1.95) p=0.04) and *GLUT-1* (median (range): 0.31 (0.01-1.42) vs 0.41 (0.19-1.56) p=0.03) in line with their inverse correlation with life-span. Unique to the comparison of the two LLI sub-groups, BPIFB4 levels were higher in healthy- as compared to non-healthy LLIs (median (range): 1.70 (0.37-78.26) vs. 0.56 (0.13-59.12) p=0.004) whereas CXCR4 expression was lower in healthy- as compared to non-healthy LLIs (median (range): 0.62 (0.07-4.94) vs. 1.38 (0.10-3.85) p=0.04) (Figure 2). The expression of CXCR4 and BPIFB4 mRNA levels were confirmed to be independently associated to the health status among LLIs in a multivariable logistic model. The odds ratio associated with a doubling expression levels was 0.62 for CXCR4 (95% CI 0.38 to 0.99, p=0.049) and 1.56 (95% CI 1.07 to 2.28, p=0.02) for BPIFB4. This means that health status among LLIs is positively associated with BPIFB4 and negatively associated with CXCR4 (OR=2.51; 95% CI: 1.31-4.82, p=0.05 when BPIFB4 doubles and CXCR4 is halved).

### CXCR4 levels identify LLIs with functional MNCs

CXCR4 expression characterizes a class of MNCs with regenerative ability that is recruited in the context of low nutrient/low oxygen conditions [20, 22]. Therefore, results of low CXCR4 levels association with health status was counterintuitive and led us to assess if MNCs from healthy-LLIs respond to CXCR4 ligand SDF-1ɑ chemo-attraction differently than MNCs of non-healthy-LLIs.

**Figure 3:**
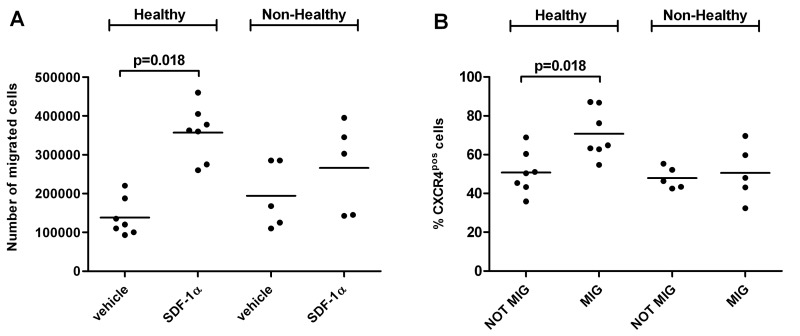

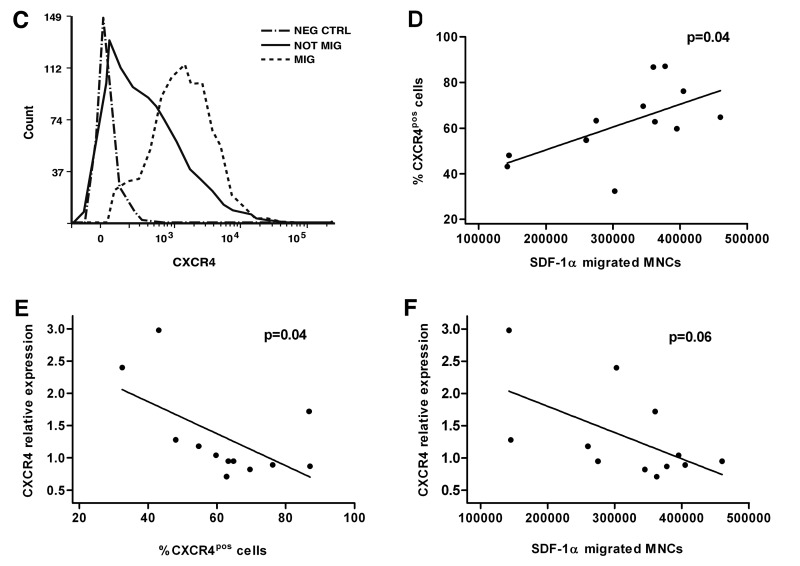
Migratory ability of LLI MNCs is impaired in non-healthy donors and associates with percentage of membrane CXCR4-positive cells (**A**) Dot plot showing results of *in vitro* migration assay performed on a separate group of healthy (N=7) and non-healthy (N=5) LLIs. Number of MNCs migrated in the lower chamber toward vehicle (migration medium containing 0.1% serum bovine albumin, BSA) or chemoattractant SDF-1ɑ (CXCL12) is shown. (**B**) Percentage of CXCR4 (SDF-1ɑ receptor)-positive MNCs was measured by flow cytometry in migrated (MIG) and not migrated (NOT MIG) harvested from the upper and lower chamber of the migration system respectively. Results of the 12 donors are shown in the dot plot. (**C**) Representative flow cytometry plot showing CXCR4^pos^ MNCs in migrated and not migrated fractions. Negative control (Neg CTRL) indicates not stained MNCs. (D-F) Association analysis of SDF-1ɑ-migrated MNCs and percentage of CXCR4^pos^ cells (P=0.04), percentage of CXCR4^pos^ cells and CXCR4 relative expression (P=0.04), and SDF-1ɑ-migrated MNCs and CXCR4 relative expression (P=0.06).

*In vitro* migration assay toward SDF-1ɑ was performed using MNCs isolated from a new group of 12 LLI donors (N=7 healthy and N=5 non-healthy) described in Table 2. Non-healthy donor MNCs were functionally impaired (Figure 3A). Flow cytometry (FACS) analyses of migrated and non-migrated cells evidenced that non-healthy donors had a lower percentage of cells expressing membrane CXCR4 (Figure 3B-C). As expected, the number of SDF-1ɑ-migrated cells was directly associated with the percentage of FACS CXCR4-positive MNCs (rho=0.6, p=0.04) pointing at the need of CXCR4 for migration (Figure 3D). Importantly, in the analysis of the association between CXCR4 RNA expression levels and CXCR4 positive migrated cells we found inverse correlation (rho=0.9, p=0.04) (Figure 3E). Similar inverse trend was observed in the correlation between CXCR4 RNA and number of migrated cells (rho=0.5, p=0.06) (Figure 3F). The CXCR4 protein abundance did not change in two LLI groups and did not show association with the RNA levels.

**Table 1:** Differential expression analyses population

Group	n	Age mean (SD)	Age range	Gender (M/F)
Controls	63	44.1 (15.5)	20-78	24/39
LLIs	45	97.4 (3.4)	92-106	16/29
Healthy-LLIs	14	99.1 (3.3)	93-106	1/13
Non-Healthy-LLIs	31	96.6 (3.3)	92-105	15/16

LLIs, all long-living individuals; Healthy-LLIs, healthy-aged long-living individuals;. Non-Healthy-LLIs, frail long-living individuals affected with diabetes, cardiovascular disease, Alzheimer's disease or dementia.

**Table 2:** Characteristic of the LLIs subjects enrolled for studying migration

Group	n	Age mean (SD)	Age range	Gender (M/F)
Healthy-LLIs	7	93.0 (3.8)	90-101	1/6
Non-Healthy-LLIs	5	92.4 (2.1)	90-95	3/2

Healthy-LLIs, healthy-aged long-living individuals; Non-Healthy-LLIs, frail long-living individuals affected with diabetes, cardiovascular disease, Alzheimer's disease or dementia.

## DISCUSSION

There is a remarkable paucity of data regarding classifiers of healthy longevity, which can help generate new hypotheses and strategies to implement wellbeing in the general population. This study investigates the association of two candidate pathways with extreme longevity and healthy longevity. One pathway was deducted from genome-wide association studies in humans and the other from data on the *C. elegans* lifespan model. Results indicate that the same set of genes in the studied pathways has a different value as a classifier of general or healthy longevity.

We have recently focused our efforts on LLIs and young controls recruited in Cilento, a rural area of southern Italy (the Southern Italian Centenarian Study-SICS) discovering the association of rs2070325 in BPIFB4 gene to LLI [4, 23]. This longevity-associated variant of the BPIFB4 gene exerts vascular protection *via* activation of the eNOS pathway and stimulates regeneration through recruitment of pro-angiogenic cells. BPIFB4 is expressed in the CD34+ sub-fraction of circulating MNCs, which is acknowledged to participate in vascular repair of ischemic tissues [4]. Interestingly, BPIFB4 was more abundantly expressed in CD34+ cells from LLIs as compared to young controls. Follow up study on serum of healthy-LLIs vs. frail-LLIs showed that protein levels correlate with extreme longevity and that the protein amount is able to discriminate between healthy- and non-healthy LLIs [5]. Here, we show that BPIFB4 mRNA levels are also elevated in the total MNC fraction from healthy LLIs as compared with non-healthy LLIs, in line with previous findings obtained in serum. On the other hand, differently from results in serum, the comparison of BPIFB4 mRNA levels in MNCs of LLIs and young controls showed no difference between the two groups. Therefore, the assessment of BPIFB4 in MNCs may help achieve a more specific determination of the health status of LLIs rather than life-span. MNCs represent a heterogeneous population with multifunctional properties. Further studies are necessary to understand if BPIFB4 expression levels in a particular cellular sub-fraction provide better indication on healthy lifespan.

The use of *C. elegans* models unveiled a complex role for HIF-1 as a longevity regulator. Indeed, several studies identified the HIF-1-mediated hypoxic response as an important longevity-promoting pathway that is distinct from insulin-like signaling and dietary restriction [24]. Interestingly, life-extending effects of HIF-1 are caused, at least in part, by the interplay between neuronal and intestinal cells. Indeed, HIF-1 activation in neurons increases serotonergic signaling, leading to higher levels of flavin-containing monooxygenase-2 in the intestine, which increased longevity [25]. However, other investigations also identified a longevity-limiting role for HIF-1 (i.e. deletion increases life span), highlighting the complex relationship between HIF and aging [24].

The present study provides a possible key of interpretation for the above discrepancies, as HIF-1 targets behaved differently as classifiers, with GLUT-1, ALDO-C, and VEGF-A being lower and miR-210 higher in the global population of LLIs. ALDO-C and miR-210 were similarly distributed between the healthy and non-healthy LLIs, making these factors promising candidates for life-span, but not for health-span determination. In contrast, VEGF-A and GLUT-1 were good classifiers of both life-span and health-span. Intriguingly, a decreased expression of CXCR4 showed a significant association with prolonged health-span and not with life-span, similarly to what observed with BPIFB4 mRNA levels. Thus, the two genes may provide a unique expressional signature of health conservation with aging independent of life-span.

CXCR4 is a seven transmembrane, G-protein-coupled, alpha-chemokine receptor specific for SDF-1ɑ. CXCR4 gene down-regulation, as observed in healthy-LLIs, did not result in reduced migratory activity following chemokine stimulation since healthy-LLIs MNCs migrated efficiently while frail-LLIs cells were impaired. Moreover, the number of cells responsive to SDF-1ɑ induced migration is proportional to the percentage of CXCR4 protein-positive cells, whereas CXCR4 RNA levels appeared to be inversely correlated with the percentage of membrane CXCR4-positive MNCs. Therefore, high CXCR4 expression in the non-healthy group may represent a futile rescue mechanism to restore the lack of membrane protein needed for migration. Interpretation of these results in chronic ischemic diseases may be even more complex when considering that a previous study from our group has shown that SDF-1-induced migration of CD45(dim)CD34+CXCR4+KDR+ MNCs was higher in patients with cardiovascular death, forecasting cardiovascular mortality independently of other validated predictors, such as age, diagnosed coronary artery disease, serum C-reactive protein, and estimated glomerular filtration rate [21]. It would be valuable to verify if silencing or enhancing BPIFB4 and CXCR4 expression in MNCs from healthy- or non-healthy-LLIs alters different MNC functions.

The observed discrepancy between different HIF targets was somewhat surprising. However, it could be due to different HIF isoforms acting on distinct targets in a cell specific manner [26]. Moreover, HIF-1 and HIF-2 transcriptional activity largely depends on the chromatin context and on the concomitant action of other transcriptional factors and cofactors that can determine the activation (or the lack of) of individual promoters and enhancers [27, 28].

Very interesting is also the association of increased miR-210 levels with LLI. One can speculate that this might be related to the cell migration stimulatory function of miR-210 [29] [12]. However, miR-210 impact on mitochondrial metabolism and reactive oxygen species production might also be important [11].

In conclusion, we here confirm the role of BPIFB4 and provide novel evidence for CXCR4 being a classifier of healthy aging. Further investigations are granted to confirm the potential of HIF targets and miR-210 to discriminate between LLIs and controls and between healthy versus non-healthy LLIs. Finally, we show that the comparison of healthy versus non-healthy LLIs is a possible strategy for the identification of genes/proteins involved in healthy aging.

## METHODS

### Patient recruitment and classification

We enrolled, by home visiting, and collected anamnestic information from a group of LLIs (individuals older than 90 years of age) from community-dwelling people based in a rural area of southern Italy (Cilento) in the attempt to identify molecular classifiers of healthy aging [4, 23]. Controls were healthy donors recruited in the same geographic area of the LLIs, with an age range between 20 and 80 years old. LLIs were sub-classified as frail or healthy by the presence of systemic diseases (which includes diabetes mellitus, cardiovascular diseases (CVDs), Alzheimer or dementia, respiratory diseases, rheumatoid arthritis). All the patients were recruited in the same period of time and the collected blood samples processed within 24h since the withdrawn.

All subjects donated blood samples for DNA study and gave written informed consent to the study, which was approved by IRCCS MultiMedica Ethical Committee, CE/CE/42/2010/LDC, protocol N. “19 2010 Cardiovascolare”. The study was conducted in accordance with the ethical principles that have their origins in the Declaration of Helsinki.

### MNC isolation

PB (15mL) was withdrawn through a forearm vein puncture and MNCs were separated on Histopaque-1077 (Sigma-Aldrich) gradient at 1200xg.

### RNA extraction and TaqMan quantitative Real Time PCR analysis

RNA was extracted from MNCs using miRNeasy kit (Qiagen) following the manufacturer's instructions. The concentration of and integrity of RNA was determined using the Nanodrop ND1000 Spectrophotometer (Thermo Scientific). RNA reverse transcription to measure miR-210 was performed with the TaqMan miR reverse transcription kit following the manufacturer's instructions (Applied Biosystems). miR expression was analyzed by QuantStudio™ 6 Flex Real-Time PCR System (Applied Biosystems) and normalized to the U6 small nucleolar RNA (snRU6). For gene expression analyses, single-strand complementary DNA (cDNA) was synthesized from 1 μg of total RNA using TaqMan Reverse Transcription reagents (Applied Biosystems). Quantitative RT-PCR was performed with the QuantStudio™ 6 Flex Real-Time PCR System (Applied Biosystems) using the following primers: 18s rRNA (forward 5′-CGCAGCTAGGAATAATGGAATAGG-3′; reverse 5′-CATGGCCTCAGTTCCGAAA-3′), AK3 (forward: 5′-CGTTGGATTCACCCTCCTAGC-3′; re-verse: 5′-GCTGGACTAACGGTTCACCA-3′), VEGF-A (forward 5′- CAACATCACCATGCAGATTATGC -3′; reverse 5′- TCGGCTTGTCACATTTTTCTTGT -3′), CXCR4 (forward: 5′-CAGTGGCCGACCTCCTCTT-3′; rever-se: 5′-GGACTGCCTTGCATAGGAAGTT-3′); ALDO-C (forward: 5′-GGCTGCCACTGAGGAGTTC-3′; rever-se: 5′-CTGCTGCTCCACCATCTTCT-3′).3′), GLUT-1 (forward: 5′-GATTGGCTCCTTCTCTGTGG-3′, reverse: 5′-TCAAAGGACTTGCCCAGTTT-3′), ADM (forward: 5′-GGAAGAGGGAACTGCGGATGT-3′, reverse: 5′-GGCATCCGGACTGCTGTCT-3′). Data were normalized to 18S ribosomal RNA as an endogenous control. For both miR and gene expression, each PCR reaction was performed in triplicate and analyses were performed by the 2ddCt method [30, 31].

### Migration assay

For MNC migration assay 3μm pore-size filter-equipped transwell chambers (Corning) coated with fibronectin 2 μg/mL were used. Cells (5×10^6^) were placed in the upper chamber and allowed to migrate toward stromal derived factor-1 alpha (SDF-1ɑ/CXCL12)(R&D) (100 ng/mL) or vehicle (control) at 37°C. The assay was stopped after 18 hours and cells that migrated to the lower chamber and cells that not migrated and remained in the upper chamber were counted.

### FACS analysis

For extracellular CXCR4 analysis, MNCs (2×10^5^ cells) migrated and not-migrated were palced in separated tubes and stained with 5 μL of CXCR4 (APC) antibody (BD Biosciences). After 15 min incubation at room temperature in the dark, cells were washed, resuspended in PBS and analyzed. For each test, percentage of positive cells was analyzed using a FACSCanto flow cytometer with the FACSDiva software (both from BD Biosciences).

### Statistical analysis

Categorical variables were compared using the chi-squared test. The Wilcoxon rank-sum test was used to compare the genes and miR expression levels between LLIs and controls. Correlation between continuous variables was evaluated using the Spearman coefficient. Multivariable logistic regression models were fitted to evaluate the independent effect of mRNA expression levels that resulted significantly associated with life-span and heath-span from the univariate analyses. Expression levels were log2-transformed prior to regression analysis since their distribution was positively skewed.

All reported p-values are two-sided. A p-value <0.05 was considered statistically significant. Statistical analyses were performed with STATA 12 software (StataCorp. 2011. Stata Statistical Software: Release 12. College Station, TX: StataCorp LP).
